# Symptom Covering

**Published:** 1896-09

**Authors:** E. L. Close

**Affiliations:** Secretary


### SYMPTOM COVERING.

(Proceedings of the Brooklyn Hahnemannian Union.)

Seven members were present at the meeting of the Brooklyn Hahnemannian Union, held March 28th, Dr. John Campbell presiding in the place of Dr. Alice Campbell, who was ill. The minutes of the preceding meeting were read, accepted, and recommended for publication, and the Secretary instructed to write a note of sympathy to the absent chairman. The subject of Dr. John Campbell's paper was "Symptom Covering," which he said was the great difficulty of the homœopathic prescription. It looks simple to correct symptoms. So thinks the family with their little case of thirds, and the so-called homœopath, who gives crude drugs on a few symptoms; but covering present symptoms alone brings no result. He gave an interesting case, where although Cocculus seemed indicated, it produced no effect, and past history elicited a suppression of skin trouble by drugs, which Sulphur brought out in three days, and chills which Ipecac still further developed. By careful treatment a cure was effected in four months. Mere symptom covering is not the beginning and end of Homœopathy.

Dr. Close suggested that *symptom studying* was also a necessity. Hahnemann devoted years to the collection, analysis, and study of symptoms, resolving them into groups; so some of the symptom-covering has been done for us.

Dr. Fincke said, pathological prescribing can be done by those who can read transactions of the societies. These many years we have found Hahnemann's view correct. We are necessitated to make a careful picture of the case, and then we must study *that* case, and decide which are the most important symptoms, giving the picture a certain character. Then to find this picture among the remedies is a serious study. When we do not get a sharply defined symptom we must gather them all and take the remedies nearest.

Dr. Baylies said, every remedy has its color, its sex, to use a figure of speech. If we cover the symptoms full enough, we cover both case and remedy. As we become more familiar with the remedies, experience enables us to see this correspondence quickly, even with a small objective point of view. He had a patient who waked often with a nervous shock beginning in the spine, pupils dilated, digestion weak, skin cool. The dilation proceeded from muscular weakness, not congestion, as it was not Belladonna. Nux was given with only transient benefit. The patient was still disturbed with a shock, but now in the abdomen, and radiating over the body. That symptom was found in Hering under "Tartar-emetic." This remedy in the 25M potency was given.

Dr. Close thought a good deal hinged on our definition of a symptom, and quoted from Dr. Wells, that a symptom is not an isolated fact, but a fact taken in connection with its associated facts and the history and surroundings that complete them. Thus the totality of symptoms means the totality of each symptom.

Dr. Fincke said the significance of a symptom is in its connection with the whole. Homœopathy is a practical art, because when in haste one cannot stop to reason out the pathological relation. His nephew fainted; he gave Aconite. In coming-to a cold sweat broke out, calling plainly for Veratrum.

Dr. Baylies related a case of caries of the tibia, caused by fracture. An ulcer refused to heal, but there were no constitutional symptoms, except that a change of weather sometimes caused a pricking sensation. Carb-veg. was being administered. Dr. Fincke suggested Symphytum, if the bones were still ununited.

Dr. Baylies said the man wanted the X ray used, but feared the electricity might not agree with him.

Dr. Fincke spoke of an experiment related in a German paper, over thirty years ago, showing light to emanate from certain crystal bodies. Now they must attenuate the air to show these beautiful colors, and then they laugh at our high potency, whose existence we prove by the effect, while their great argument is only experiment. Everything leads to Homœopathy, as that is a part of universal force.

Dr. Lutze referred to the different effects of the same force, as when gravitation causes a stone to fall and a balloon to rise. Dr. John Campbell suggested, *so* we can trace the divinity of Homœopathy. Dr. Close thought the conclusions of science are approaching higher planes, and Dr. Fincke answered that the more they get these manifestations the more they stick to matter. They will not go down upon their knees before the Almighty.

Dr. Campbell said that Hahnemann refers to another force than medicinal, as when cold water warms we recognize the law.

Dr. Close related the case of a man of seventy-five, where a severe cold brought him in a week very low, and one night found him delirious, alternately praying and talking sensually, singing and muttering; high fever and involuntary stools. In the morning he was nearly unconscious, eyes glazed, tongue dry, stiff, and brown, limbs stiff—almost dead. Stramonium$^{200}$ in water, repeated till improvement came. Next morning he was found much better, slept all night, and had called for something to eat, and the second day he was sitting up.

Dr. Fincke told a peculiar experience where a neighbor called him to see his daughter who had been confined and was very sick, apparently unconscious and dying. Rhus was given, and the next day she said, "Why have you called me back? I'm sorry—I was far away." Then the other doctor came with his drugs and the next day she was dead.

Dr. Baylies gave a most complicated case of a woman who six weeks after confinement had scarlet fever and Bright's disease. When he saw her after three months she was taking seven different medicines, including a powerful preparation of Digitalis, and having her bladder washed out several times a day with Boracic-acid, the urine was twenty-five per cent. pus. She had symptoms of a grade of meningitis. Sulphur millionth gave her a quiet night and she became from day to day more composed. In five days there was but twelve per cent. pus in the urine. After a steady improvement came a telephone message not to call any more as his wife was well.

Dr. Close told of a child of six with diphtheria who was roused every fifteen minutes, either for a drug, milk punch, swab or spray. It died on the eighth day.

Dr. Fincke related the case of one child where the parents feared it would have diphtheria and began injecting Anti-toxine and the child died on the fourth day.

Dr. Campbell gave a case of miscarriage where twenty-five things were tried in a few hours, such as Turpentine on the abdomen and tampons, and said the so-called homœopath who was giving this treatment must have been a real "Symptom-coverer," giving a remedy for every symptom!

The meeting adjourned after electing Dr. Anna Carman chairman for April.

E. L. Close, *Secretary.*

